# Mass Cytometry Identifies Expansion of T-bet^+^ B Cells and CD206^+^ Monocytes in Early Multiple Sclerosis

**DOI:** 10.3389/fimmu.2021.653577

**Published:** 2021-05-04

**Authors:** Laura Couloume, Juliette Ferrant, Simon Le Gallou, Marion Mandon, Rachel Jean, Nadège Bescher, Helene Zephir, Gilles Edan, Eric Thouvenot, Aurelie Ruet, Marc Debouverie, Karin Tarte, Patricia Amé, Mikael Roussel, Laure Michel

**Affiliations:** ^1^ INSERM, Unité Mixte de Recherche U1236, Université Rennes, Etablissement Français du Sang Bretagne, LabEx IGO, Rennes, France; ^2^ Pole Biologie-CHU Rennes, 2 rue Henri Le Guilloux, Rennes, France; ^3^ CHU Lille, CRCSEP Lille, Univ Lille, Lille, France; ^4^ Neurology Department, Rennes Clinical Investigation Centre, Rennes University Hospital-Rennes University-INSERM, Rennes, France; ^5^ Department of Neurology, Nimes University Hospital, Nimes, France; ^6^ Institut de Génomique Fonctionnelle, UMR5203, Inserm 1191, Université de Montpellier, Montpellier, France; ^7^ Université de Bordeaux, Bordeaux, France; ^8^ Neurocentre Magendie, INSERM U1215, Bordeaux, France; ^9^ CHU de Bordeaux, Department of Neurology, Bordeaux, France; ^10^ Nancy University Hospital, Department of Neurology, Nancy, France; ^11^ Université de Lorraine, APEMAC, Nancy, France

**Keywords:** biomarker, multiple sclerosis, mass cytometry, immunology, B cells, monocytes

## Abstract

Multiple sclerosis (MS) is an immune-driven demyelinating disease of the central nervous system. Immune cell features are particularly promising as predictive biomarkers due to their central role in the pathogenesis but also as drug targets, even if nowadays, they have no impact in clinical practice. Recently, high-resolution approaches, such as mass cytometry (CyTOF), helped to better understand the diversity and functions of the immune system. In this study, we performed an exploratory analysis of blood immune response profiles in healthy controls and MS patients sampled at their first neurological relapse, using two large CyTOF panels including 62 markers exploring myeloid and lymphoid cells. An increased abundance of both a T-bet-expressing B cell subset and a CD206^+^ classical monocyte subset was detected in the blood of early MS patients. Moreover, T-bet-expressing B cells tended to be enriched in aggressive MS patients. This study provides new insights into understanding the pathophysiology of MS and the identification of immunological biomarkers. Further studies will be required to validate these results and to determine the exact role of the identified clusters in neuroinflammation.

## Introduction

Multiple sclerosis (MS) is a frequent chronic inflammatory demyelinating disease of the central nervous system (CNS) and is a leading cause of nontraumatic disability in young adults. Although its precise etiology remains to be identified, immune cells have been proposed as one of the main players in MS, especially during the early phase of the disease (1). Indeed, immunotherapies targeting lymphocytes and monocytes have a beneficial effect on MS disease activity. Besides T cells, that are well-known as major players in MS, B cells have been recently pointed out for their diverse pathogenic effects: as pro inflammatory cytokine- and antibody-secreting cells, but also as antigen-presenting cells (2). These cells have also probably an important role in the progressive form of MS by contributing to the formation of tertiary ectopic lymphoid follicles inside the CNS (3, 4). Besides the adaptive immune system, the innate arm of the immune system (including monocytes, macrophages, dendritic cells, and microglia) has been investigated in mouse models of MS and was shown to be involved in MS pathogenesis. In particular, activated microglia and macrophages are the main cells described in MS active lesions (5) and myeloid cells act as antigen-presenting cells and effector cells in the context of neuroinflammation (6).

Immune cell features are thus particularly promising from a biomarker perspective due to their central role in MS pathogenesis but also as drug targets. Although the phenotypic and functional analysis of immune cells is an appealing strategy for understanding immune-mediated disease processes, immune cell profiling has currently no impact in clinical practice. Recently, high-resolution approaches, such as mass cytometry (CyTOF), helped to better understand diversity and function of the immune system and to highlight potential targets for novel therapies. CyTOF, combined with high-dimensional analysis, is a robust method to identify numerous and poorly-described cell subsets from heterogeneous populations, including in the autoimmune context (7, 8).

In this study, we deeply analyzed peripheral blood mononuclear cell (PBMCs) samples from drug-naïve early MS patients compared to age and sex-paired controls. We herein describe an increased abundance of a T bet-expressing B cell subset and a CD206^+^ classical monocyte subset in the blood of MS patients opening new immunological pathways to investigate.

## Material and Methods

### Cohort

This study was registered and approved by the Ethics Committee of Rennes Hospital (notice n° 20.05) and was registered in clinicaltrials.gov (NCT04510350). All participants provided written informed consent. Blood samples were obtained from 11 early MS patients and 8 age and sex-paired healthy controls (HC).

MS patients included in this work participated to the OFSEP (Observatoire Français de la Sclérose en Plaques) cohort. Participating centers were Rennes, Lille, Nancy, Nimes and Bordeaux.

Inclusion criteria were: i) age > 18 years old, ii) MS diagnosis according to McDonald 2017 criteria at the last visit (9), iii) sampled during their first neurological episode, and iv) with at least one visit/year during the follow-up. Progressive MS patients were excluded. At time of PBMC sampling, all MS patients included were drug-naïve and so had never been treated by a disease modifying therapy (DMT).

Patients were classified into two groups according to the severity of the disease. “Aggressive MS” patients were defined as displaying a disease activity under disease modifying therapy (DMT) (minimum 6 months of treatment), or as having experienced two or more relapses within one year with residual disability associated with radiological activity (defined by the occurrence of at least one new T2 lesion or at least one gado-enhanced lesion on MRI). “Non-aggressive MS” patients were defined as having no disease activity during the follow-up, or as having disease activity without fulfilling the criteria for “aggressive MS”. Disease activity was defined by the occurrence of at least one relapse and/or the occurrence of radiological activity.

PBMCs were collected and frozen in each center during the first neurological episode before transfer to Rennes Hospital for analysis. Frozen PBMCs from healthy controls were obtained from a biobanking from Rennes Hospital (NCT03744351).

### Mass Cytometry

Antibodies (**Supplementary Table 1** for Lymphoid panel and **Supplementary Table 2** for Myeloid panel) were purchased either already metal-tagged (Fluidigm) or in purified form. For the last ones, they were labeled using the Maxpar Antibody Labeling Kit (Fluidigm), titrated, and stored at 4°C in Ab-stabilization buffer (Candor Bioscience). Cell labeling was performed as previously described (10). Briefly, cells were stained 5 minutes in RPMI supplemented with 0,5 μM Cisplatin Cell-ID™ (Fluidigm, San Francisco, CA) in RPMI 1640 before washing with 10% FBS in RPMI 1640. Cell were resuspended in 80μl of 0,5% BSA in PBS. Then, 60μl of each surface staining cocktail (lymphoid or myeloid) were added to 40μl of resuspended cells. After staining, cells were washed in 0,5% BSA in PBS before fixation and permeabilization with the transcription factor staining buffer set (Miltenyi, Bergisch-Gladbach, Germany). Then 60μl of each intracellular staining cocktail (lymphoid or myeloid), were added to 40μl of resuspended cells in Perm Buffer. After intracellular staining, cells were washed twice before staining in DNA intercalator solution (2.5% Paraformaldehyde, 1:3200 Cell-ID™ Intercalator-Ir (Fluidigm, San Francisco, CA) in PBS). Samples were cryopreserved at -80°C until acquisition on Helios™ System (Fluidigm, San Francisco, CA).

### Data Analysis

**Figure 1:**
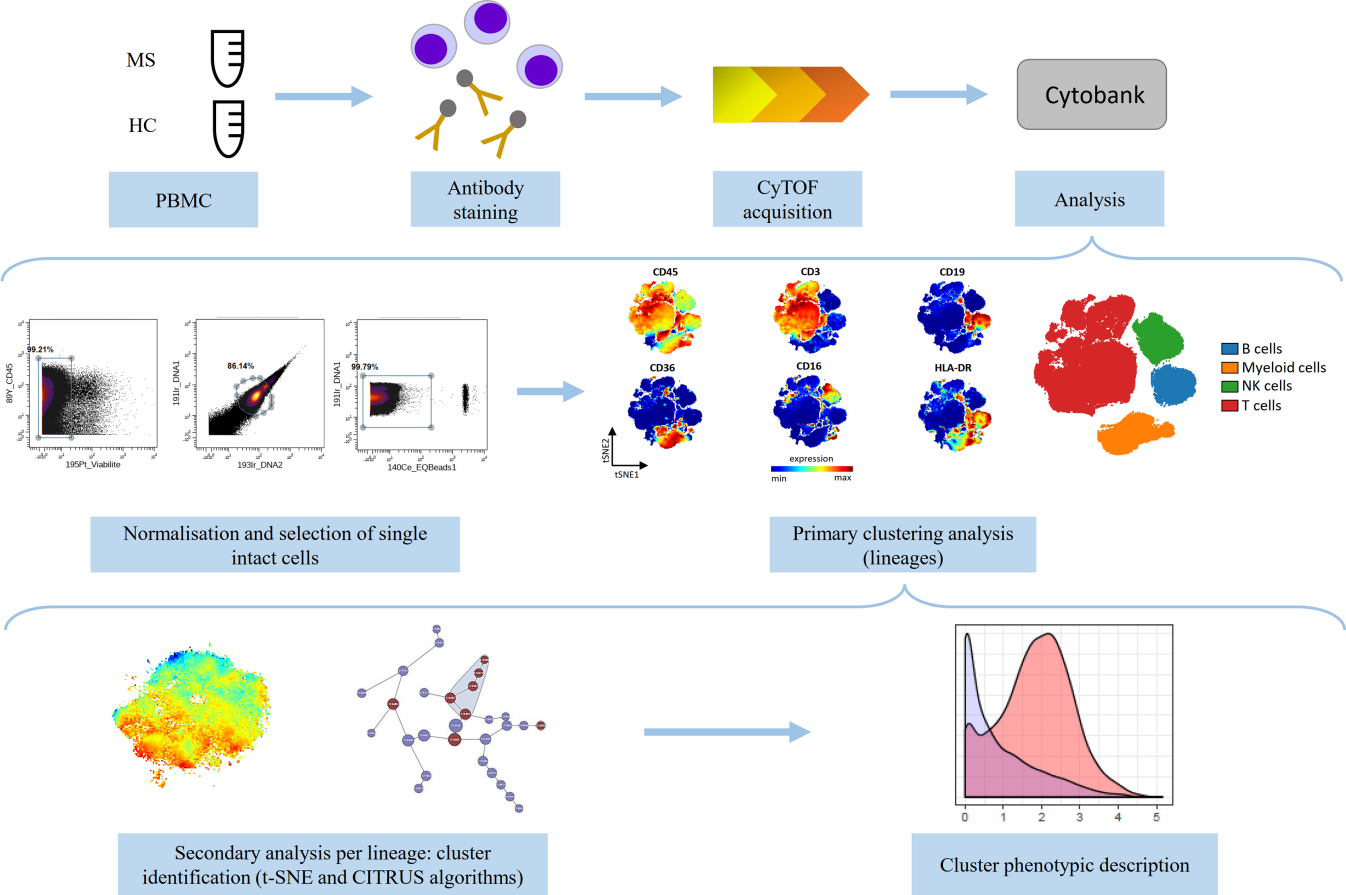
Schematic representation of the experimental design of the study. Peripheral blood mononuclear cells (PBMCs) from multiple sclerosis (MS, n=11) patients and healthy controls (HC, n=8) were divided into two equal parts and stained with two antibody panels (Lymphoid panel and Myeloid panel, **Supplementary Tables 1**, **2**) and acquired on the CyTOF instrument. The data were normalized and single intact cells were manually selected. Primary analysis was performed to identify lineage cell subsets and secondary analysis was performed to describe phenotypic alterations per lineage between MS and HC (using t-SNE and CITRUS algorithms). Phenotypic description of the identified clusters was performed.

The experimental design of the study is summarized in **Figure 1**.

After acquisition, intrafile signal drift was normalized and.fcs files were obtained using CyTOF software. To diminish batch effects, all files were normalized on EQ Beads (Fluidigm Sciences) using the premessa R package (https://github.com/ParkerICI/premessa). Files were then uploaded to the Cytobank cloud-based platform (Cytobank, Inc.). For all files, live single cells were selected by applying a gate on DNA1 vs. DNA2 followed by a gate on DNA1 vs. Cisplatin, then beads were removed by applying a gate on the beads channel (Ce140Di) vs. DNA1.

Each file was first analyzed using viSNE, based upon the Barnes–Hut implementation of t-SNE. The analysis was performed based on the event count of the file or on the maximum total events allowed by Cytobank. The following parameters were used: perplexity = 30; iterations = 5000; theta = 0.45. For the lymphoid panel, all 36 channels were selected and for the myeloid panel, all 33 channels were selected. The two panels included CD45, CD3, CD19, CD16, CD36, and HLA-DR, allowing selection of B-, T-, NK-, and myeloid cells. Then, we performed a t-SNE algorithm per lineage, to compare immune profile of MS patients and HC. Equal downsampling was performed, based on the lowest event count in all files. The following parameters were used: perplexity = 30; iterations = 5000; theta = 0.45.

We then applied a clustering method using the CITRUS algorithm. CITRUS proceeds through an unsupervised hierarchical clustering to identify clusters of cellular populations within the overall dataset, and then calculate biologically relevant features of these clustered populations. We performed the CITRUS algorithm on the previous viSNE results for each lineage, using all panel channels, and the following parameters: association model = SAM (Significance Analysis of Microarrays); mode of cluster characterization = abundance; event sampling = equal; minimum cluster size = 5%; FDR (False Discovery Rate) = 1%.

Finally, phenotypic description of identified clusters was performed.

Data generated during this study can be shared upon reasonable request.

### Statistical Analysis

Statistical analyses were performed with GraphPad Prism 8.4.2 Software. For comparisons, normality tests were first performed, and then Student’s t test or Mann Whitney test were used when appropriate. A p value < 0.05 was considered statistically significant.

## Results

### Patients

Analyses were performed on PBMC samples isolated from 11 MS patients and 8 HC. The demographic, clinical, and radiological data of MS patients and HC are detailed in **Table 1**. Gender and age did not differ between patients and controls [sex (% women): MS = 72.7, HC = 62.5, p > 0.99; age (years ± SD): MS = 31.4 ± 9.2, HC = 33.4 ± 9.5, p = 0.65]. Mean duration of follow-up was 30.8 ± 12.1 months. At last follow up, six patients were classified as « non-aggressive MS » and five as « aggressive MS ».

**Table 1:** Characteristics of MS patients and healthy controls at baseline.

	Controls	MS (Total)	Aggressive MS	Non-aggressive MS
n	8	11	5	6
**Demographic characteristics**				
Age in years (mean ± SD)	33.4 ± 9.5	31.4 ± 9.2	31.6 ± 8.3	31.2 ± 10.6
Women (%)	5 (62.5)	8 (72.7)	3 (60)	5 (83.3)
**Clinical characteristics**				
Delay between the first relapse and the sample collection in days (mean ± SD)	–	53 ± 43	52.4 ± 25.9	53.5 ± 56.3
Mains symptoms at first relapse				
*Optic neuritis (%)*	–	4 (36.4)	2 (40)	2 (33.3)
*Motor symptoms (%)*	–	3 (27.3)	1 (20)	2 (33.3)
*Sensory symptoms (%)*	–	3 (27.3)	1(20)	2 (33.3)
*Vestibular/Cochlear symptoms (%)* *Oculomotor symptoms (%)*	- -	1 (9.1) 1 (9.1)	1 (20) 1 (20)	0 (0) 0 (0)
EDSS Score at baseline (mean ± SD)	–	2.1 ± 1.5	2.2 ± 1.7	2 ± 1.5
**Radiological characteristics**				
Presence of at least one gadolinium-enhancing lesion on the MRI at baseline (%)	–	3 (27.3)	0 (0)	3 (50)

MS, multiple sclerosis; SD, standard deviation; EDSS, expanded disability status scale.

### Identification of Lineage Cell Subsets

We characterized PBMCs from MS patients and HC using two separate mass cytometry panels exploring lymphoid and myeloid subsets; respectively (**Supplementary Table 1** for Lymphoid panel and **Supplementary Table 2** for Myeloid panel). We first compared the immune cell composition of PBMCs between MS patients and HC. We found that T cell abundance was lower in MS patients than in HC (MS=54.1%, HC=64.4%, p=0.002), whereas the proportion of the other cell lineages (B cells, NK cells, and myeloid cells) was not significantly modulated between the two groups (data not shown).

### Phenotypic Alterations in the Lymphoid Compartment in Patients With Early MS

**Figure 2:**
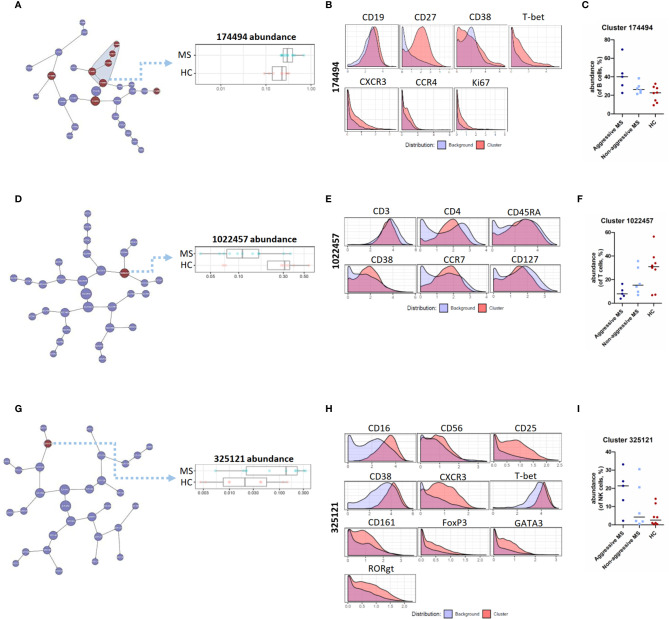
CITRUS analysis of mass cytometry data from PBMC of MS patients and HC – B, T, NK cell lineages. **(A)** Visual representation of unsupervised hierarchical clustering of B cells and visualization of the clusters that are part of the significant results (in red). Abundance of the cluster 174494 (of B cells) in MS patients and HC. **(B)** Expression of CD19, CD27, CD38, T-bet, CXCR3, CCR4, and Ki67 on cells from cluster 174494 as compared to all other (background) B cells. **(C)** Abundance of cluster 174494 (of B cells, %) in « aggressive MS » patients, « non-aggressive MS » patients and HC. **(D)** Visual representation of unsupervised hierarchical clustering of T cells and visualization of the cluster that are part of the significant results (in red). Abundance of the cluster 1022457 (of T cells) in MS patients and HC. **(E)** Expression of CD3, CD4, CD45RA, CD38, CCR7, CD127 measured on cells from cluster 1022457 as compared to all other (background) T cells. **(F)** Abundance of cluster 1022457 (of T cells, %) in « aggressive MS » patients, « non-aggressive MS » patients and HC. **(G)** Visual representation of unsupervised hierarchical clustering of NK cells and visualization of the cluster that are part of the significant results (in red). Abundance of the cluster 325121 (of NK cells) in MS patients and HC. **(H)** Expression of CD16, CD56, CD25, CD38, CXCR3, T-bet, CD161, FoxP3, GATA3, and RORgt measured on cells from cluster 325121 as compared to all other (background) NK cells. **(I)** Abundance of cluster 325121 (of NK cells, %) in « aggressive MS » patients, « non-aggressive MS » patients and HC.

Using CITRUS algorithm, we first analyzed B cell subsets and highlighted 8 clusters with a significantly different abundance between MS patients and HC, including the parent cluster 174494 (**Figure 2A**). This memory B cell cluster (CD19^+^CD27^+^CD38^-^) had a higher expression of the T-bet transcription factor. These T-bet^+^ B cells also expressed migration and proliferation markers (CXCR3, CCR4, and Ki67) in a more pronounced way than other B cell clusters (**Figure 2B**). This cluster showed a trend towards a higher abundance in “aggressive MS” patients than in “non-aggressive MS” patients, even if the low number of patients precluded any definitive conclusion (**Figure 2C**). Cluster 174403 that identified activated naïve B cells (CD19^+^CD27^-^CD38^+^CXCR5^+^HLA-DR^++^CCR4^+^) had also a significantly higher abundance in MS patients than in HC (data not shown) whereas two clusters of naïve B cells (CD19^+^CD27^-^CD38^+^) were reduced in MS patients compared to HC, including the cluster 174486 expressing low levels of CXCR3 and CCR4, and the cluster 174493 identifying naïve B cells with a low activation level (lower expression of CD44 and CD45RA than other clusters of B cells) (data not shown).

Concerning T cell subsets, we identified the “activated naïve” T cell cluster 1022457, expressing CD3, CD4, CD45RA, CD38, CCR7, and CD127 as significantly reduced in MS patients compared with HC (**Figures 2D, E**). Interestingly, this cluster showed a trend towards a lower abundance in “aggressive MS” patients than in “non-aggressive MS” patients (**Figure 2F**).

Finally, one cluster of NK cells, cluster 325121, had a significantly higher abundance in MS patients than in HC (**Figure 2G**). These cells were CD56^dim^ CD16^hi^ cytotoxic NK cells, and were positive for CD25, CD38, CXCR3, T-bet, and CD161, corresponding to “pro-inflammatory” NK cells. This cluster also expressed FoxP3, GATA3, and RORgt transcription factors in a more pronounced way than NK cells from other clusters (**Figure 2H**). Again, it showed a trend towards a higher abundance in “aggressive MS” patients than in “non-aggressive MS” patients (**Figure 2I**).

As a whole, we identified specific B cell, T cell, and NK cell clusters deregulated in MS patients with a tendency towards an amplification of these alterations in aggressive MS.

### Phenotypic Alterations of the Myeloid Compartment in Patients With Early MS

**Figure 3:**
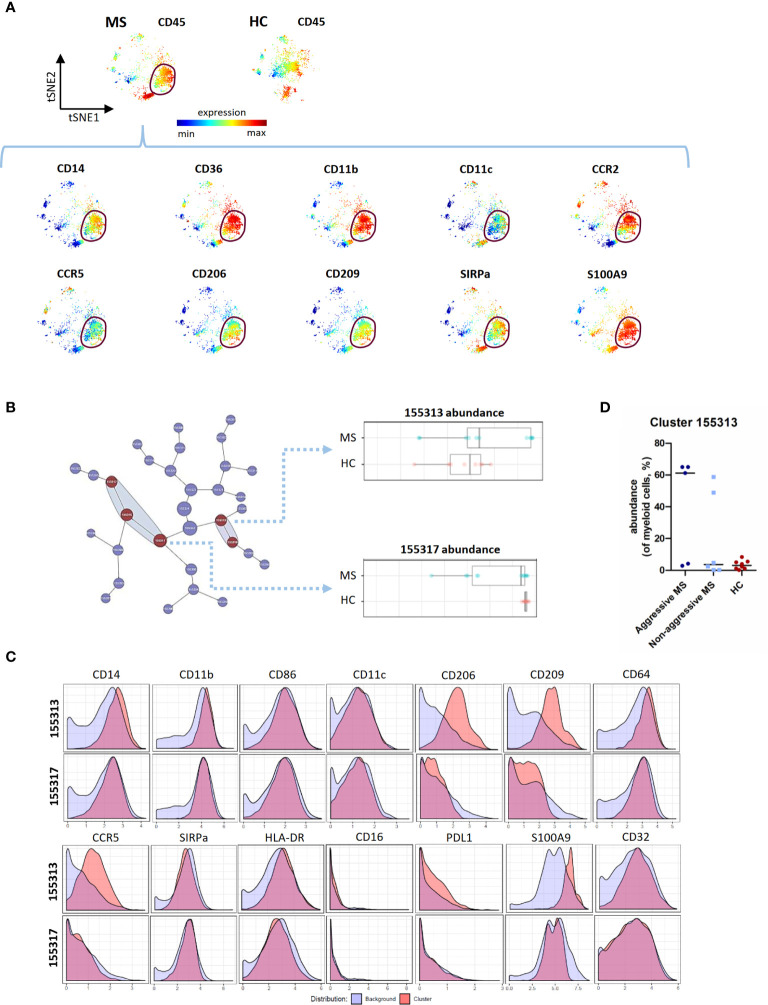
CITRUS analysis of mass cytometry data from PBMC of MS patients and HC - Myeloid cell lineage. **(A)** Representative t-SNE maps, of a MS patient and a HC, regarding myeloid cells lineage. The t-SNE maps were generated based on expression levels of all markers of the myeloid panel (**Supplementary Table 2**). Description of a myeloid subset in a MS patient (circled). Each dot represents one cell and the color spectrum represents individual marker-expression levels (red : high expression; blue : low expression). **(B)** Visual representation of unsupervised hierarchical clustering of myeloid cells and visualization of the clusters that are part of the significant results (in red). Abundance of the cluster 155313 and 155317 (of myeloid cells) in MS patients and HC. **(C)** Expression of CD14, CD11b, CD86, CD11c, CD206, CD209, CD64, CCR5, SIRPa, HLA-DR, CD16, PD-L1, S100A9 and CD32 measured on cells from cluster 155313 and 155317 as compared to all other (background) myeloid cells. **(D)** Abundance of cluster 155313 (of myeloid cells, %), in « aggressive MS » patients, « non aggressive MS » patients and HC.

We then performed viSNE analysis of the myeloid lineage. We detected, by manual gating, a peculiar myeloid subpopulation in 5 out of 11 MS patients and in none of 8 HC (chi square test = 0.026). This population expressed CD14, CD36, CD11b, CD11c, CCR2, CCR5, CD206, CD209, SIRPa, and S100A9 (**Figure 3A**). This subset was also identified (cluster 155313) using CITRUS as enriched in MS patients compared to HC, and corresponded to a subset of classical monocytes with an increased expression of pro-inflammatory (CD86, CD64, CD32) and regulatory (CD206, CD209, PD-L1) markers (**Figures 3B, C**). Using viSNE and CITRUS approaches, this subset was found in the same 3 “aggressive MS” patients, and 2 “non-aggressive MS” patients whereas it was not detected in HC **(** **Figure 3D** **).**

Contrary to the cluster 155313, cluster 155317 had a significantly lower abundance in MS patients than in HC (**Figure 3B**). Compared to cluster 155313, it expressed low level of CD206, CD209, CCR5, and S100A9 (**Figure 3C**), corresponding to classical monocytes with pro-inflammatory but not regulatory markers.

## Discussion

MS is a complex and heterogeneous disease with a highly variable disease course, reflecting, among other things, the interplay between genetic background and environmental factors. Even if many progresses have been made concerning the implication of the different subsets of immune cells in the pathological mechanisms, the crosstalk between the different immune cell subsets remains unclear.

Here, using extensive immune cell profiling exploring both adaptive and innate immune system, we describe a subset of T-bet^+^ B cells particularly abundant in early MS patients, especially in the aggressive form of MS. We also found a decrease of a naïve T cell subset with activation markers, and an increase of a pro-inflammatory NK cell subset in the blood of MS patients. Finally, we found the presence of a CD206^+^ classical monocyte subset in the blood of 5/11 untreated early MS patients.

Multiple lines of evidence have recently pinpointed the key contribution of B lymphocytes to MS pathogenesis. In the relapsing remitting form of MS, the administration of B Cell Depleting Therapy (BCDT), using anti-CD20 monoclonal antibodies (mAb), was demonstrated to clinically and radiologically improve MS patients (11, 12). The therapeutic benefit of the BCDT is thought to result from the elimination of B cells with pathogenic properties (13–15). In the peripheral blood of MS patients, pathogenic subsets of B cells were recently identified and characterized as pro-inflammatory GM-CSF- (16) and TNFα-secreting B cells (17, 18). In our study, we show that CD27^+^ CD38^-^ T-bet^+^ B cells, that express CXCR3, CCR4 and Ki67, are significantly increased in early MS patients, with a trend towards a higher abundance in “aggressive MS” patients. In Human, T-bet expression can be induced in B cells by IL-27, IFNγ, or IL-21 (19). *In vivo*, T-bet is critical for maintaining antigen specific memory of IgG2a B cells (20). In autoimmunity, B-cell specific loss of T-bet in murine models of systemic lupus erythematosus (SLE) results in reduced disease manifestation (21), whereas CD11c^hi^T-bet^+^ B cells are overrepresented in SLE patients and correlated with clinical manifestations (22). These cells are poised to generate plasmablasts producing autoreactive antibodies (22). In MS, a recent study suggests that T-bet-expressing B cells are recruited in patient CNS (23). In our study, these T-bet^+^ B cells seemed to be more represented in aggressive MS patients. The exact mechanism by which T-bet-expressing B cells are involved in neuroinflammation remains to be explored, and further studies are needed to link them to disease severity.

In this study, we also show that CD4^+^ CD38^+^ CD45RA^+^ CD127^+^ CCR7^+^ T cells are decreased in early MS patients. Even if CD38 is considered as an activation marker, its expression on “naïve” T cells has already been reported (24). Recently, CD4^+^ T cells with a similar phenotype were studied in HC. These cells were low proliferative, had a bias towards IL-13 secretion, and showed responsiveness to IL-7 (25). Among NK cells, we found an increase of an activated pro-inflammatory CD56^dim^CD16^hi^ CD161^+^ subset in MS patients. In fact, a recent study suggests that CD161 defines a subset of pro-inflammatory NK cells that may contribute to inflammatory disease pathogenesis (26). The exact implication of these two last lymphocyte subsets in neuroinflammation remains to be precisely defined.

In MS, the different populations of myeloid cells, including monocytes, macrophages, microglia, and dendritic cells, have prominent roles in the pathogenesis as antigen-presenting cells (in periphery and inside the CNS) but also as effector cells (inside the CNS) (6). There are only few studies concerning circulating myeloid cells from MS patients, and data are not consistent (27, 28). Here we report an increased abundance of a myeloid cell subset with a phenotype corresponding to classical monocytes with pro-inflammatory markers (markers associated with M1 macrophage polarization, CD86, CD64, CD32), regulatory markers (markers associated with M2 macrophage polarization, CD206, CD209, PD-L1) and a high expression of S100A9. Very interestingly, the majority of activated macrophages in active MS lesions were shown to display a mixed pro-inflammatory and regulatory status (29). These results are consistent with those of a more recent study, in which iNOS/CD206 double positive macrophages were detected in all chronic active MS lesions examined with a higher frequency in the MS lesion center (30). It is tempting to speculate that the classical monocyte cluster highlighted in our study corresponds to circulating precursors of these CNS macrophages with intermediate activation status and their interest as biomarker related to MS severity deserves further evaluation.

By now, only few studies have used mass cytometry to deeply analyze in an unbiased manner PBMCs from MS patients. In particular, Böttcher et al. identified an increased abundance of CCR7^+^ and IL6^+^ T cells in early MS patients, whereas CD141^hi^ IRF8^hi^ CXCR3^+^ CD68^-^ dendritic cells were decreased (31). Galli et al. identified an expanded T helper cell subset characterized by the expression of GM-CSF and CXCR4 (32). Finally, Marsh-Wakefield et al. used a B-cell focused mass cytometry panel to compare peripheral IgG3^+^ B cells of MS patients with inactive or active stages of disease (33). Overall, considering mass cytometry analyses of PBMCs in MS patients, the literature gathers only few studies using different panels and variable inclusion criteria, resulting in data that are difficult to compare and explaining the non-redundant results obtained.

Our study also has some limitations. The major one is the low number of MS patients and HC, sometimes resulting in data with high variability within the cohort. Moreover, the small number of patients did not allow us to make robust conclusions concerning the analysis between the different MS patient subgroups. Of note, enrolled patients were highly selected in order to constitute the most homogeneous cohort, including drug-naïve MS patients selected at the stage of the first neurological relapse. Nevertheless, these preliminary data will have to be confirmed using larger studies.

So, even if the number of patients was small, they were highly selected (first relapse, no DMT) and well characterized. Moreover, one of the strengths of our study is the use of two large antibody panels exploring both lymphoid and myeloid compartments. This pilot study opens the way to a bigger study with a high number of well-characterized patients to robustly compare immune profile of different MS patient subgroups according to disease severity, and so validate these potential biomarkers.

Using comprehensive immune profiling of PBMCs at the single cell level and unsupervised approach, we have been able to detect subsets of immune cells (T-bet^+^ B cells, CD206^+^ classical monocytes, pro-inflammatory NK cells) significantly increased in our cohort of early untreated MS patients compared to controls. These results open a path of biomarkers discovery using the power of high dimensional single cell techniques.

## Data Availability Statement

The original contributions presented in the study are included in the article/**Supplementary Material**. Further inquiries can be directed to the corresponding author.

## Ethics Statement

The studies involving human participants were reviewed and approved by Ethics Committee of Rennes Hospital. The patients/participants provided their written informed consent to participate in this study.

## Observatoire Français de la Sclérose en Plaques

The following contributors, who are listed in alphabetical order, contributed to the work of Observatoire Français de la Sclérose en Plaques:

**OFSEP investigators**
CISCO project
**List of OFSEP investigators**
*(Steering Committee, Principal investigators, Biology group, Imaging group)*
***Steering Comittee***
**Romain Casey**, *PhD, Observatoire français de la sclérose en plaques (OFSEP), Centre de coordination national, Lyon/Bron, France;*
**François Cotton**, *MD, Hospices civils de Lyon, Hôpital Lyon sud, Service d’imagerie médicale et interventionnelle, Lyon/Pierre-Bénite, France;*
**Jérôme De Sèze**, *MD, Hôpitaux universitaire de Strasbourg, Hôpital de Hautepierre, Service des maladies inflammatoires du système nerveux – neurologie, Strasbourg, France;*
**Pascal Douek**, *MD, Union pour la lutte contre la sclérose en plaques (UNISEP), Ivry-sur-Seine*, *France;*
**Francis Guillemin**, *MD, CIC 1433 Epidémiologie Clinique, Centre hospitalier régional universitaire de Nancy, Inserm et Université de Lorraine, Nancy, France;*
**David Laplaud**, *MD, Centre hospitalier universitaire de Nantes, Hôpital nord Laennec, Service de neurologie, Nantes/Saint-Herblain,  France;*
**Christine Lebrun-Frenay**, *MD, Centre hospitalier universitaire de Nice, Université Nice Côte d’Azur, Hôpital Pasteur, Service de neurologie, Nice, France;*
**Lucilla Mansuy**, *Hospices civils de Lyon, Département de la recherche clinique et de l’innovation, Lyon, France;*
**Thibault Moreau**, *MD, Centre hospitalier universitaire Dijon Bourgogne, Hôpital François Mitterrand, Service de neurologie, maladies inflammatoires du système nerveux et neurologie générale, Dijon, France;*
**Javier Olaiz**, *PhD, Université Claude Bernard Lyon 1, Lyon ingéniérie projets, Lyon, France;*
**Jean Pelletier**, *MD, Assistance publique des hôpitaux de Marseille, Centre hospitalier de la Timone, Service de neurologie et unité neuro-vasculaire, Marseille, France;*
**Claire Rigaud-Bully**, *Fondation Eugène Devic EDMUS contre la sclérose en plaques, Lyon, France;*
**Bruno Stankoff**, *MD, Assistance publique des hôpitaux de Paris, Hôpital Saint-Antoine, Service de neurologie, Paris, France;*
**Sandra Vukusic**, *MD, Hospices civils de Lyon, Hôpital Pierre Wertheimer, Service de neurologie A, Lyon/Bron, France;*
**Hélène Zephir**, *MD, Centre hospitalier universitaire de Lille, Hôpital Salengro, Service de neurologie, Lille, France;*
***Investigators***
**Romain Marignier**, *MD, Hospices civils de Lyon, Hôpital Pierre Wertheimer, Service de neurologie A, Lyon/Bron, France;*
**Marc Debouverie**, *MD, Centre hospitalier régional universitaire de Nancy, Hôpital central, Service de neurologie, Nancy, France;*
**Gilles Edan**, *MD, Centre hospitalier universitaire de Rennes, Hôpital Pontchaillou, Service de neurologie, Rennes, France;*
**Jonathan Ciron**, *MD, Centre hospitalier universitaire de Toulouse, Hôpital Purpan, Service de neurologie inflammatoire et neuro-oncologie, Toulouse, France;*
**Aurélie Ruet**, *MD, Centre hospitalier universitaire de Bordeaux, Hôpital Pellegrin, Service de neurologie, Bordeaux, France;*
**Nicolas Collongues**, *MD, Hôpitaux universitaire de Strasbourg, Hôpital de Hautepierre, Service des maladies inflammatoires du système nerveux – neurologie, Strasbourg, France;*
**Catherine Lubetzki**, *MD, Assistance publique des hôpitaux de Paris, Hôpital de la Pitié-Salpêtrière, Service de neurologie, Paris, France;*
**Hélène Zephir**, *MD, Centre hospitalier universitaire de Lille, Hôpital Salengro, Service de neurologie, Lille, France;*
**Pierre Labauge**, *MD, Centre hospitalier universitaire de Montpellier, Hôpital Gui de Chauliac, Service de neurologie, Montpellier, France;*
**Gilles Defer**, *MD, Centre hospitalier universitaire de Caen Normandie, Service de neurologie, Hôpital Côte de Nacre, Caen, France;*
**Mikaël Cohen**, *MD, Centre hospitalier universitaire de Nice, Université Nice Côte d’Azur, Hôpital Pasteur, Service de neurologie, Nice, France;*
**Agnès Fromont**, *MD, Centre hospitalier universitaire Dijon Bourgogne, Hôpital François Mitterrand, Service de neurologie, maladies inflammatoires du système nerveux et neurologie générale, Dijon, France;*
**Sandrine Wiertlewsky**, *MD, Centre hospitalier universitaire de Nantes, Hôpital nord Laennec, Service de neurologie, Nantes/Saint-Herblain, France;*
**Eric Berger**, *MD, Centre hospitalier régional universitaire de Besançon, Hôpital Jean Minjoz, Service de neurologie, Besançon, France;*
**Pierre Clavelou**, *MD, Centre hospitalier universitaire de Clermont-Ferrand, Hôpital Gabriel-Montpied, Service de neurologie, Clermont-Ferrand, France;*
**Bertrand Audoin**, *MD, Assistance publique des hôpitaux de Marseille, Centre hospitalier de la Timone, Service de neurologie et unité neuro-vasculaire, Marseille, France;*
**Claire Giannesini**, *MD, Assistance publique des hôpitaux de Paris, Hôpital Saint-Antoine, Service de neurologie, Paris, France;*
**Olivier Gout**, *MD, Fondation Adolphe de Rothschild de l’œil et du cerveau, Service de neurologie, Paris, France;*
**Eric Thouvenot**, *MD, Centre hospitalier universitaire de Nîmes, Hôpital Carémeau, Service de neurologie, Nîmes, France;*
**Olivier Heinzlef**, *MD, Centre hospitalier intercommunal de Poissy Saint-Germain-en-Laye, Service de neurologie, Poissy, France;*
**Abdullatif Al-Khedr**, *MD, Centre hospitalier universitaire d’Amiens Picardie, Site sud, Service de neurologie, Amiens, France;*
**Bertrand Bourre**, *MD, Centre hospitalier universitaire Rouen Normandie, Hôpital Charles-Nicolle, Service de neurologie, Rouen, France;*
**Olivier Casez**, *MD, Centre hospitalier universitaire Grenoble-Alpes, Site nord, Service de neurologie, Grenoble/La Tronche, France;*
**Philippe Cabre**, *MD, Centre hospitalier universitaire de Martinique, Hôpital Pierre Zobda-Quitman, Service de Neurologie, Fort-de-France, France;*
**Alexis Montcuquet**, *MD, Centre hospitalier universitaire Limoges, Hôpital Dupuytren, Service de neurologie, Limoges, France;*
**Alain Créange**, *MD, Assistance publique des hôpitaux de Paris, Hôpital Henri Mondor, Service de neurologie, Créteil, France;*
**Jean-Philippe Camdessanché**, *MD, Centre hospitalier universitaire de Saint-Étienne, Hôpital Nord, Service de neurologie, Saint-Étienne, France;*
**Justine Faure**, *MD, Centre hospitalier universitaire de Reims, Hôpital Maison-Blanche, Service de neurologie, Reims, France;*
**Aude Maurousset**, *MD, Centre hospitalier régional universitaire de Tours, Hôpital Bretonneau, Service de neurologie, Tours, France;*
**Ivania Patry**, *MD, Centre hospitalier sud francilien, Service de neurologie, Corbeil-Essonnes, France;*
**Karolina Hankiewicz**, *MD, Centre hospitalier de Saint-Denis, Hôpital Casanova, Service de neurologie, Saint-Denis, France;*
**Corinne Pottier**, *MD, Centre hospitalier de Pontoise, Service de neurologie, Pontoise, France;*
**Nicolas Maubeuge**, *MD, Centre hospitalier universitaire de Poitiers, Site de la Milétrie, Service de neurologie, Poitiers, France;*
**Céline Labeyrie**, *MD, Assistance publique des hôpitaux de Paris, Hôpital Bicêtre, Service de neurologie, Le Kremlin-Bicêtre, France;*
**Chantal Nifle**, *MD, Centre hospitalier de Versailles, Hôpital André-Mignot, Service de neurologie, Le Chesnay, France;*
***Biology group***
**Patrick Gelé** *, CRB/CIC1403, Centre de Biologie Pathologie Génétique, Lille, France*
**Mireille Desille-Dugast** *, CRB, Laboratoire de Cytogénétique et Biologie Cellulaire, CHU Pontchaillou, Rennes, France ;*
**Céline Loiseau** *, CRB, Laboratoire de cytogénétique, CHU de Nîmes, Nîmes, France;*
**Julien Jeanpetit** *, Centre de Ressources Biologiques Plurithématique (CRB-P), Bordeaux Biothèques Santé (BBS), Pôle de Biologie et de Pathologie, CHU de Bordeaux, Bordeaux, France;*
**Sandra Lomazzi** *, CRB Lorrain- CHRU Nancy, Vandoeuvre-les-Nancy, France;*
**David Laplaud**, *Centre hospitalier universitaire de Nantes, Hôpital nord Laennec, Service de neurologie, Nantes/Saint-Herblain,  France;*
**Eric Thouvenot**, *Centre hospitalier universitaire de Nîmes, Hôpital Carémeau, Service de neurologie, Nîmes, France;*
**Guillaume Brocard**, *Observatoire français de la sclérose en plaques (OFSEP), Centre de coordination national, Lyon/Bron, France;*
**Romain Casey**, *Observatoire français de la sclérose en plaques (OFSEP), Centre de coordination national, Lyon/Bron, France;*
**Nathalie Dufay** *, NeuroBioTec, Hôpital Neurologique Pierre Wertheimer, Hospices Civils de Lyon, Lyon/Bron, France;*
**Caroline Barau** *, Laboratoire de la PRB, Centre d’Investigation Clinique (CIC), Groupe Hospitalier Henri Mondor, Créteil, France;*
**Shaliha Bechoua**, *Etablissement Français du Sang, Service Biothèque-CRB, Dijon, France;*
**Gilda Belrose**, *Centre de Ressources Biologiques de la Martinique (CeRBiM), CHU de Martinique Pierre ZOBDA-QUITMAN, Fort-de-France, France;*
**Juliette Berger**, *CRB Auvergne - CHU Estaing, Clermont-Ferrand, France;*
**Marie-Pierrette Chenard** *, CRB, UF 6337, Département de Pathologie, Hôpital de Hautepierre, Hôpitaux Universitaires de Strasbourg, Strasbourg, France;*
**Esther Dos Santos** *, Service de Biologie médicale, Poissy, France ;*
**Arianna Fiorentino** *, CRB HUEP-SU, Faculté de médecine site Saint Antoine, Paris, France;*
**Sylvie Forlani** *, Banque ADN & Cellules-ICM U1127, PRB, GH Pitié-Salpêtrière,Paris, France;*
**Géraldine Gallot** *, CRB, UF 7296, CHU de Nantes, Hôtel Dieu, Institut de biologie, Nantes, France;*
**Michèle Grosdenier** *, EFS, CHU de Poitiers, Poitiers, France ;*
**Yves-Edouard Herpe** *, Biobanque de Picardie - CHU Amiens-Picardie, Amiens, France;*
**Caroline Laheurte** *, Etablissement Français du Sang, Besançon, France;*
**Hélène Legros**, *CHU Caen Normandie, Caen, France;*
**Sylvain Lehmann** *, CHU Saint Eloi, IRMB, Biochimie Protéomique Clinique, Montpellier, France;*
**Philippe Lorimier** *, Centre de Ressources Biologiques, Institut de Biologie et de Pathologie, CHU Albert Michallon, Grenoble, France;*
**Mikael Mazighi** *, Fondation Ophtalmologique Adolphe de Rothschild, Centre de Ressources Biologiques, Paris, France;*
**Samantha Montagne** *, CHRU Bretonneau, CRB, EFS, Tours, France.*
**Bénédicte Razat** *, CRB Toulouse Bio Ressources, Toulouse, France;*
**Noémie Saut** *, Service d'Hématologie Biologique, CHU Timone adultes, Marseille, France ;*
**Emilie Villeger** *, CRBIoLim, CHU Dupuytren, Limoges, France;*
**Kevin Washetine** *, CHU de Nice, Hôpital Pasteur 1, Nice, France;*
***Imaging group***
**Olivier Outteryck**, *MD, CHRU Lille, Consultations de neurologie D, Lille, France;*
**Jean-Pierre Pruvo** *, MD, CHRU Lille, Service de radiologie, Lille, France;*
**Elise Bannier** *, PHD Institut de Recherche en Informatique et Systèmes Aléatoires, Rennes, France;*
**Jean-Christophe Ferre** *, MD, CHU Rennes, Service de radiologie, Rennes, France;*
**Thomas Tourdias** *, MD, CHU Bordeaux, Service de radiologie, Bordeaux, France;*
**Vincent Dousset** *, MD, CHU Bordeaux, Service de radiologie, Bordeaux, France;*
**Rene Anxionnat** *, MD, CHU Nancy, Service de radiologie, Nancy, France;*
**Roxana Ameli**, *MD, Hospices civils de Lyon, Service de radiologie, Lyon, France;*
**Arnaud Attye** *, MD, CHU de Grenoble, Service de radiologie, Grenoble, France;*
**Douraied Bensalem** *, MD, CHU Brest, Service de radiologie, Brest, France;*
**Marie-Paule Boncoeur-Martel** *, MD, CHU Limoges, Service de radiologie, Limoges, France;*
**Fabrice Bonneville** *, MD, CHU Toulouse Purpan, Service de radiologie, Toulouse, France;*
**Claire Boutet** *, MD, CHU Saint-Etienne, Service de radiologie, Saint-Etienne, France;*
**Jean-Christophe Brisset** *, PHD Median technologies, Valbonne, France;*
**Frédéric Cervenanski** *, PHD CREATIS, Villeurbanne, France;*
**Béatrice Claise** *, MD, CHU Clermont-Ferrand, Service de radiologie, Clermont-Ferrand, France;*
**Olivier Commowick** *, I PHD NRIA, Rennes, France;*
**Jean-Marc Constans** *, MD, CHU Amiens – Picardie, Service de radiologie, Amiens, France;*
**Pascal Dardel** *, MD, CH Chambéry, Service de radiologie, Chambéry, France;*
**Hubert Desal** *, MD, CHU Nantes, Service de radiologie, Nantes, France;*
**Françoise Durand-Dubief** *, MD, Hospices civils de Lyon, Service de Neurologie, Lyon, France;*
**Alina Gaultier** *, MD, CHU Nantes, Service de radiologie, Nantes, France;*
**Emmanuel Gerardin** *, MD, CHU Rouen, Service de radiologie, Rouen, France;*
**Tristan Glattard** *, PHD CREATIS, Villeurbanne, France;*
**Sylvie Grand** *, MD, CHU de Grenoble, Service de radiologie, Grenoble, France;*
**Thomas Grenier** *, PHD CREATIS, Villeurbanne, France;*
**Rémy Guillevin** *, MD, CHR Poitiers, Service de radiologie, Poitiers, France;*
**Charles Guttmann** *, MD, Harvard Medical School, Boston, USA;*
**Alexandre Krainik** *, MD, CHU Grenoble Alpes, Service de radiologie, Grenoble, France;*
**Stéphane Kremer** *, MD, CHU Strasbourg, Service de radiologie, Strasbourg, France;*
**Stéphanie Lion** *, Centre de coordination national de l’OFSEP, Lyon/Bron, France;*
**Nicolas Menjot de Champfleur** *, MD, CHU Montpellier, Service de radiologie, Montpellier, France;*
**Lydiane Mondot**, *MD, CHU Nice, Service de radiologie, Nice, France;*
**Nadya Pyatigorskaya** *, MD, ICM, Service de radiologie, Paris, France;*
**Sylvain Rabaste** *, MD, Hospices civils de Lyon, Service de radiologie, Lyon, France;*
**Jean-Philippe Ranjeva** *, MD, APHM - CHU Marseille Timone, Service de radiologie, Marseille, France;*
**Jean-Amédée Roch** *, MD, Hôpital privé Jean Mermoz, Service de radiologie, Lyon, France;*
**Jean Claude Sadik** *, MD, Fondation A. de Rothschild, Service de radiologie, Paris, France;*
**Dominique Sappey-Marinier** *, MD, Hospices civils de Lyon, Service de radiologie, Lyon, France;*
**Julien Savatovsky** *, MD, Fondation A. de Rothschild, Service de radiologie, Paris, France;*
**Jean-Yves Tanguy** *, MD, CH Angers, Service de radiologie, Angers, France;*
**Ayman Tourbah** *, MD, Hôpital Raymond Poincaré, Service de Neurologie, Garches, France;*

## Author Contributions

MR and LM: study conception and design. LC, MR and LM: literature search. HZ, GE, ET, AR and MD: included patients. LC, JF, SG, MM, RJ and NB: acquisition of data. LC, JF, MR and LM: data analysis and interpretation. LC: figures. LC, LM and MR: drafting the article. LC, JF, SG, MM, RJ, NB, HZ, GE, ET, AR, MD, KT, PA, MR and LM: critical revision of the article and final approval of the version to be published. All authors contributed to the article and approved the submitted version.

## Funding

This study was partly funded by Roche. The funder was not involved in the study design, collection, analysis, interpretation of data, the writing of this article or the decision to submit it for publication.

## Conflict of Interest

The authors declare that the research was conducted in the absence of any commercial or financial relationships that could be construed as a potential conflict of interest.

## References

[1] Lazibat I Rubinić Majdak M Županić S. Multiple Sclerosis: New Aspects of Immunopathogenesis. Acta Clin Croat (2018) 57(2):352–61. 10.20471/acc.2018.57.02.17 PMC6532002 30431730

[2] Wanleenuwat P Iwanowski P. Role of B Cells and Antibodies in Multiple Sclerosis. Mult Scler Relat Disord (2019) 36:101416. 10.1016/j.msard.2019.101416 31577986

[3] Serafini B Rosicarelli B Magliozzi R Stigliano E Aloisi F. Detection of Ectopic B-cell Follicles With Germinal Centers in the Meninges of Patients With Secondary Progressive Multiple Sclerosis. Brain Pathol (2004) 14(2):164–74. 10.1111/j.1750-3639.2004.tb00049.x PMC8095922 15193029

[4] Magliozzi R Howell O Vora A Serafini B Nicholas R Puopolo M. Meningeal B-cell Follicles in Secondary Progressive Multiple Sclerosis Associate With Early Onset of Disease and Severe Cortical Pathology. Brain (2007) 130(Pt 4):1089–104. 10.1093/brain/awm038 17438020

[5] Lassmann H. Multiple Sclerosis Pathology. Cold Spring Harb Perspect Med (2018) 8:a028936. 10.1101/cshperspect.a028936 PMC5830904 29358320

[6] Mishra MK Yong VW. Myeloid Cells - Targets of Medication in Multiple Sclerosis. Nat Rev Neurol (2016) 12(9):539–51. 10.1038/nrneurol.2016.110 27514287

[7] Guo R Zhang T Meng X Lin Z Lin J Gong Y. Lymphocyte Mass Cytometry Identifies a CD3-CD4+ Cell Subset With a Potential Role in Psoriasis. JCI Insight (2019) 4(6):e125306. 10.1172/jci.insight.125306 PMC6483065 30747724

[8] Rao DA Gurish MF Marshall JL Slowikowski K Fonseka CY Liu Y. Pathologically Expanded Peripheral T Helper Cell Subset Drives B Cells in Rheumatoid Arthritis. Nature (2017) 542(7639):110–4. 10.1038/nature20810 PMC5349321 28150777

[9] Thompson AJ Banwell BL Barkhof F Carroll WM Coetzee T Comi G. Diagnosis of Multiple Sclerosis: 2017 Revisions of the McDonald Criteria. Lancet Neurol (2018) 17(2):162–73. 10.1016/S1474-4422(17)30470-2 29275977

[10] Ferrant J Le Gallou S Manson G Genebrier S Mourcin F Tarte K. High-Dimensional Phenotyping of Human Myeloid-Derived Suppressor Cells/Tumor-Associated Macrophages in Tissue by Mass Cytometry. Methods Mol Biol (2021) 2236:57–66. 10.1007/978-1-0716-1060-2_6 33237540

[11] Hauser SL Waubant E Arnold DL Vollmer T Antel J Fox RJ. B-Cell Depletion With Rituximab in Relapsing-Remitting Multiple Sclerosis. N Engl J Med (2008) 358(7):676–88. 10.1056/NEJMoa0706383 18272891

[12] Hauser SL Bar-Or A Comi G Giovannoni G Hartung H-P Hemmer B. Ocrelizumab Versus Interferon Beta-1a in Relapsing Multiple Sclerosis. N Engl J Med (2017) 376(3):221–34. 10.1056/NEJMoa1601277 28002679

[13] Bar-Or A Fawaz L Fan B Darlington PJ Rieger A Ghorayeb C. Abnormal B-cell Cytokine Responses a Trigger of T-cell-mediated Disease in MS? Ann Neurol (2010) 67(4):452–61. 10.1002/ana.21939 20437580

[14] Piccio L Naismith RT Trinkaus K Klein RS Parks BJ Lyons JA. Changes in B- and T-lymphocyte and Chemokine Levels With Rituximab Treatment in Multiple Sclerosis. Arch Neurol (2010) 67(6):707–14. 10.1001/archneurol.2010.99 PMC2918395 20558389

[15] Barr TA Shen P Brown S Lampropoulou V Roch T Lawrie S. B Cell Depletion Therapy Ameliorates Autoimmune Disease Through Ablation of IL-6-Producing B Cells. J Exp Med (2012) 209(5):1001–10. 10.1084/jem.20111675 PMC3348102 22547654

[16] Li R Rezk A Miyazaki Y Hilgenberg E Touil H Shen P. Proinflammatory GM-CSF-Producing B Cells in Multiple Sclerosis and B Cell Depletion Therapy. Sci Transl Med (2015) 7(310):310ra166. 10.1126/scitranslmed.aab4176 26491076

[17] Duddy M Niino M Adatia F Hebert S Freedman M Atkins H. Distinct Effector Cytokine Profiles of Memory and Naive Human B Cell Subsets and Implication in Multiple Sclerosis. J Immunol (2007) 178(10):6092–9. 10.4049/jimmunol.178.10.6092 17475834

[18] Michel L Chesneau M Manceau P Genty A Garcia A Salou M. Unaltered Regulatory B-cell Frequency and Function in Patients With Multiple Sclerosis. Clin Immunol (2014) 155(2):198–208. 10.1016/j.clim.2014.09.011 25267439

[19] Knox JJ Myles A Cancro MP. T-Bet+ Memory B Cells: Generation, Function, and Fate. Immunol Rev (2019) 288(1):149–60. 10.1111/imr.12736 PMC6626622 30874358

[20] Wang NS McHeyzer-Williams LJ Okitsu SL Burris TP Reiner SL McHeyzer-Williams MG. Divergent Transcriptional Programming of Class-Specific B Cell Memory by T-bet and RORα. Nat Immunol (2012) 13(6):604–11. 10.1038/ni.2294 PMC3362691 22561605

[21] Rubtsova K Rubtsov AV Thurman JM Mennona JM Kappler JW Marrack P. B Cells Expressing the Transcription Factor T-bet Drive Lupus-Like Autoimmunity. J Clin Invest (2017) 127(4):1392–404. 10.1172/JCI91250 PMC5373868 28240602

[22] Wang S Wang J Kumar V Karnell JL Naiman B Gross PS. Il-21 Drives Expansion and Plasma Cell Differentiation of Autoreactive CD11chiT-Bet+ B Cells in SLE. Nat Commun (2018) 9(1):1758. 10.1038/s41467-018-03750-7 29717110 PMC5931508

[23] van Langelaar J Rijvers L Janssen M Wierenga-Wolf AF Melief M-J Siepman TA. Induction of Brain-Infiltrating T-Bet-Expressing B Cells in Multiple Sclerosis. Ann Neurol (2019) 86(2):264–78. 10.1002/ana.25508 PMC6771938 31136008

[24] Dianzani U Funaro A DiFranco D Garbarino G Bragardo M Redoglia V. Interaction Between Endothelium and CD4+CD45RA+ Lymphocytes. Role of the Human CD38 Molecule. J Immunol (1994) 153(3):952–9. 7913116

[25] Scalzo-Inguanti K Plebanski M. CD38 Identifies a Hypo-Proliferative IL-13-secreting CD4+ T-cell Subset That Does Not Fit Into Existing Naive and Memory Phenotype Paradigms. Eur J Immunol (2011) 41(5):1298–308. 10.1002/eji.201040726 21469087

[26] Kurioka A Cosgrove C Simoni Y van Wilgenburg B Geremia A Björkander S. CD161 Defines a Functionally Distinct Subset of Pro-Inflammatory Natural Killer Cells. Front Immunol (2018) 9:486. 10.3389/fimmu.2018.00486 29686665 PMC5900032

[27] Waschbisch A Schröder S Schraudner D Sammet L Weksler B Melms A. Pivotal Role for CD16+ Monocytes in Immune Surveillance of the Central Nervous System. J Immunol (2016) 196(4):1558–67. 10.4049/jimmunol.1501960 26746191

[28] Gjelstrup MC Stilund M Petersen T Møller HJ Petersen EL Christensen T. Subsets of Activated Monocytes and Markers of Inflammation in Incipient and Progressed Multiple Sclerosis. Immunol Cell Biol (2018) 96(2):160–74. 10.1111/imcb.1025 PMC5836924 29363161

[29] Vogel DYS Vereyken EJF Glim JE Heijnen PDAM Moeton M van der Valk P. Macrophages in Inflammatory Multiple Sclerosis Lesions Have an Intermediate Activation Status. J Neuroinflamm (2013) 10:35. 10.1186/1742-2094-10-35 PMC3610294 23452918

[30] Giles DA Washnock-Schmid JM Duncker PC Dahlawi S Ponath G Pitt D. Myeloid Cell Plasticity in the Evolution of Central Nervous System Autoimmunity. Ann Neurol (2018) 83(1):131–41. 10.1002/ana.25128 PMC5876132 29283442

[31] Böttcher C Fernández-Zapata C Schlickeiser S Kunkel D Schulz AR Mei HE. Multi-Parameter Immune Profiling of Peripheral Blood Mononuclear Cells by Multiplexed Single-Cell Mass Cytometry in Patients With Early Multiple Sclerosis. Sci Rep (2019) 9(1):19471. 10.1038/s41598-019-55852-x 31857644 PMC6923404

[32] Galli E Hartmann FJ Schreiner B Ingelfinger F Arvaniti E Diebold M. GM-CSF and CXCR4 Define a T Helper Cell Signature in Multiple Sclerosis. Nat Med (2019) 25(8):1290–300. 10.1038/s41591-019-0521-4 PMC6689469 31332391

[33] Marsh-Wakefield F Ashhurst T Trend S McGuire HM Juillard P Zinger A. IgG3+ B Cells are Associated With the Development of Multiple Sclerosis. Clin Transl Immunol (2020) 9(5):e01133. 10.1002/cti2.1133 PMC7190396 32355561

