# Supplementary material for: Mass Cytometry Identifies Expansion of T-bet+ B Cells and CD206+ Monocytes in Early Multiple Sclerosis
Source: Front Immunol. 2021 May 4;12:653577. doi: 10.3389/fimmu.2021.653577 (PMC8129576; doi:10.3389/fimmu.2021.653577)
Supplement: Supplementary file 1 [file Table_1.docx]

**Supplementary Table 1 : Antibodies used for mass cytometry staining - Lymphoid Panel**

| Target | Clone | Company | Isotope | Localization |
| --- | --- | --- | --- | --- |
| CD8 | RPA-T8 | BioLegend | 141Pr | Cell-surface |
| CD19 | HIB19 | Biolegend | 142Nd | Cell-surface |
| HLA-DR | 10.1 | Biolegend | 143Nd | Cell-surface |
| CD4 | RPA-T4 | BioLegend | 144Nd | Cell-surface |
| CD16 | B73.1 | Biolegend | 145Nd | Cell-surface |
| CD25 | BC96 | BioLegend | 146Nd | Cell-surface |
| CD38 | HIT2 | BioLegend | 147Sm | Cell-surface |
| CXCR3 (CD183) | G025H7 | BioLegend | 148Nd | Cell-surface |
| FOXP3 | 259D/C7 | BD Biosciences | 149Sm | Intracellular |
| CD7 | CD7-6B7 | BioLegend | 150Nd | Cell-surface |
| GATA3 | TWAJ | Invitrogen | 151Eu | Intracellular |
| CCR7 (CD197) | G043H7 | BioLegend | 152Sm | Cell-surface |
| CCR6 (CD196) | G034E3 | BioLegend | 153Eu | Cell-surface |
| CD27 | O323 | BioLegend | 154Sm | Cell-surface |
| CD36 | 5-271 | Biolegend | 155Gd | Cell-surface |
| CD10 | HI10a | BioLegend | 156Gd | Cell-surface |
| CD117 | 104D2 | BioLegend | 158Gd | Cell-surface |
| CCR4 | L291H4 | BioLegend | 159Tb | Cell-surface |
| CD161 | HP-3G10 | BioLegend | 160Gd | Cell-surface |
| CXCR5 (CD185) | J252D4 | BioLegend | 161Dy | Cell-surface |
| CD3 | UCHT1 | Biolegend | 162Dy | Cell-surface |
| RORgt | AFKJS-9 | eBioscience | 163Dy | Intracellular |
| CRTH2 (CD294) | BM16 | BioLegend | 164Dy | Cell-surface |
| LAG3 | 7H2C65 | BioLegend | 165Ho | Cell-surface |
| CTLA4 (CD152) | L3D10 | BioLegend | 166Er | Intracellular |
| PD1 | EH12.2H7 | BioLegend | 167Er | Cell-surface |
| Tim3 | F38-2E2 | BioLegend | 168Er | Cell-surface |
| CD127 | A019D5 | BioLegend | 169Tm | Cell-surface |
| BCL6 | k112-91 | BD Biosciences | 170Er | Intracellular |
| T-bet | 4B10 | BioLegend | 171Yb | Intracellular |
| CD45RO | UCHL1 | BioLegend | 172Yb | Cell-surface |
| CD56 | HCD56 | BioLegend | 173Yb | Cell-surface |
| CD45RA | HI100 | BioLegend | 174Yb | Cell-surface |
| Ki67 | Ki-67 | BioLegend | 175Lu | Intracellular |
| CD44 | BJ18 | BioLegend | 176Yb | Cell-surface |
| CD45 | HI30 | Fluidigm | 89Y | Cell-surface |
