# Supplementary material for: Mass Cytometry Identifies Expansion of T-bet+ B Cells and CD206+ Monocytes in Early Multiple Sclerosis
Source: Front Immunol. 2021 May 4;12:653577. doi: 10.3389/fimmu.2021.653577 (PMC8129576; doi:10.3389/fimmu.2021.653577)
Supplement: Supplementary file 2 [file Table_2.docx]

**Supplementary Table 2 : Antibodies used for mass cytometry staining - Myeloid Panel**

| Target | Clone | Company | Isotope | Localization |
| --- | --- | --- | --- | --- |
| CD326 (EpCAM) | 9C4 | Biolegend | 141Pr | Cell-surface |
| CD19 | HIB19 | Biolegend | 142Nd | Cell-surface |
| HLA-DR | 10.1 | Biolegend | 143Nd | Cell-surface |
| CD16 | B73.1 | Biolegend | 145Nd | Cell-surface |
| CD64 | L243 | Biolegend | 146Nd | Cell-surface |
| CD11c | 3.9 | Biolegend | 147Sm | Cell-surface |
| CD33 | WM53 | Biolegend | 148Nd | Cell-surface |
| CD209 | 9E9A8 | Biolegend | 149Sm | Intracellular |
| CD14 | M5E2 | Biolegend | 150Nd | Cell-surface |
| CD123 (IL-3R) | 6H6 | Biolegend | 151Eu | Cell-surface |
| CD21 | Bu32 | Biolegend | 152Sm | Cell-surface |
| CD192 (CCR2) | K036C2 | Biolegend | 153Eu | Cell-surface |
| CD163 | GHI/61 | Biolegend | 154Sm | Cell-surface |
| CD36 | 5-271 | Biolegend | 155Gd | Cell-surface |
| CD86 | IT2.2 | Biolegend | 156Gd | Cell-surface |
| CD169 | 7-239 | Biolegend | 158Gd | Cell-surface |
| CD274 (PD-L1) | 29E.2A3 | Biolegend | 159Tb | Cell-surface |
| CD106 | EPR5047 | abcam | 161Dy | Intracellular |
| CD3 | UCHT1 | Biolegend | 162Dy | Cell-surface |
| CD49a | TS2/7 | Biolegend | 163Dy | Cell-surface |
| CD80 | 2D10 | Biolegend | 165Ho | Cell-surface |
| CD1a | HI149 | Biolegend | 167Er | Cell-surface |
| CX3CR1 | 2A9-1 | Biolegend | 168Er | Cell-surface |
| CD32 | FUN-2 | Biolegend | 169Tm | Cell-surface |
| CD54 | HA58 | Biolegend | 170Er | Cell-surface |
| CD195 (CCR5) | J418F1 | Biolegend | 171Yb | Cell-surface |
| CD206 (MMR) | 15-2 | Biolegend | 172Yb | Cell-surface |
| S100A9 | A15105J | Biolegend | 173Yb | Intracellular |
| CD45RA | HI100 | Biolegend | 174Yb | Cell-surface |
| CD172a (SIRPa) | 15-414 | Biolegend | 175Lu | Cell-surface |
| CD68 | Y1/82A | Biolegend | 176Yb | Intracellular |
| CD11b (Mac-1) | ICRF44 | Fluidigm | 209Bi | Cell-surface |
| CD45 | HI30 | Fluidigm | 89Y | Cell-surface |
